# Lectures on Subjects Connected with Clinical Medicine, Comprising Diseases of the Heart

**Published:** 1847-01-01

**Authors:** 


### Lectures on Subjects connected with Clinical Medicine, comprising Diseases of the Heart. By *P. M. Latham*, M.D. Physician Extraordinary to the Queen, and late Physician of St. Bartholomew's Hospital. Vol. II. pp. 419. London: Longman and Co., 1846.

In the Number of this Journal for July 1845, will be found an ample review of the first volume of these Lectures. We have now to continue the analysis of the valuable information which they contain on Diseases of the Heart. Although the important subject of endocardial and pericardial inflammation occupied much of our attention on the former occasion, there still remain a few points in the history of these morbid states, that call for notice.

The *prognosis* in cases of Endocarditis and Pericarditis is—provided the diseases have been sufficiently early detected, and at the same time judiciously treated—unquestionably favourable, if we have respect only to the present restoration of the patient. But what the proportion of cases is in which—although the patient so recovers his health as to be able to resume, without inconvenience, his wonted occupation—the foundation is laid of future cardiac mischief, it is by no means easy to determine. Dr. Latham tells us that of 90 cases of acute Endocarditis and Pericarditis, the result of Rheumatism, observed by him, death took place in not more than three. But of the remaining 87, it was in 17 only that he could feel anything like an assurance of a perfect recovery having taken place—*perfect*, as indicated by the cessation of all abnormal sounds or murmurs. With respect to the other 70 cases, although the patients were discharged from the hospital as recovered, in none had the heart entirely lost all the auscultatory signs of having suffered; and we need scarcely say that the circumstance of a persistent cardiac murmur, remaining after an attack of Carditis, indubitably demonstrates that some degree of structural lesion, however slight, has been left behind.

But the danger of cardiac inflammation is not always proportionate to its severity or to the amount of local injury inflicted on the central organ of the circulation. The state of the patient's general constitution, and the condition of other visceral organs, as well as of the fluids of the body, more especially of the blood itself, have much to do with the chances of recovery in each individual case. Let us briefly note a few of the fatal casualties or accidents, which are observed to occur every now and then in the clinical experience of carditic disease.

1. In some patients, at the very time when the symptoms of recovery may be reasonably expected to take place, a sudden prostration of the nervous system occasionally supervenes, and then there may be such alternate rallying and sinking that, for weeks together, a fatal issue is daily looked for. Yet the patient will less frequently die than recover from this alarming state;—not indeed with a perfect recovery, but so far at least as regards his immediate safety: the heart retains more or less of the footprints of the disease.

2. In other cases, under the same circumstances, a state of extreme anæmia occurs. These, too, not unfrequently recover eventually. The restoration, however, is necessarily very tedious, and full of anxiety.

When either of these conditions, whether of nervous exhaustion or of sanguineous impoverishment, is present, œdema of the feet and ankles often makes its appearance. Nay, the dropsy sometimes becomes general. But even then, the effused fluid may become absorbed under the use of appropriate remedies, and every trace of disease, save of a slight cardiac lesion, may be ultimately dissipated.

3. Symptoms of most grave disorder in some portion of the cerebro-spinal axis may occur in a patient, after his having passed through the acute stage of pericardial or endocardial inflammation, and suddenly carry him off, when everything promises the fair prospect of his speedy recovery.

"Coincident with symptoms referable to the endocardium or pericardium, in one case there has been maniacal delirium, in another epileptic or tetanic convulsion, in another chorea, in another coma, in another fatuity. The patients have died, and dissection has found the brain healthy, and the spinal marrow healthy, and the endocardium and the pericardium alone inflamed. Now, have all the experiments that were ever done or perpetrated upon living animals given intimation of an influence like this, proceeding from the heart to the brain, and from the heart to the spinal marrow; Has not disease here been our teacher?

"All these affections of the brain and spinal marrow, coming on in the course of inflammation of the heart, should be carefully watched and ministered to from the least to the greatest. Wild delirium, epileptic, or tetanic convulsion, chorea, coma, fatuity, are the greatest and the rarest; and mutterings, reveries, transitions from torpor to excitement, subsultus, are the least and the most frequent. But they are all akin one to another. The least may mount up to the greatest, and the greatest run down to the least.

"Moreover, where any of these have been during the progress of the disease, and the patient has survived, they are liable to be continued or to recur during its reparation. Or they may then arise for the first time, as if they took advantage of the weakness and exhaustion of the nervous system." P. 19.

These attacks of Cerebral mischief, occurring during the continuance of, or the convalescence from, cardiac inflammation, very generally prove fatal. Their pathological history is far from having, as yet, been satisfactorily made out. The necroscopic appearances of the encephalic contents have been, in not a few instances, obscure and inconclusive. How strikingly was this true in the case of suppurative inflammation of the muscular substance of the heart, quoted by Dr. Latham from an early volume of the Medico-Chirurgical Transactions:—"A boy, twelve years of age, was in perfect health on Saturday night and dead on the following Tuesday afternoon at two o'clock. He had, in the opinion of all who saw him, the severest inflammation of the brain. The attack was sudden with great heat and frequency of pulse. He had delirium and convulsions, and pointed to his forehead as the seat of his pain. At length he sank into a state of insensibility and died. Upon dissection, not a vestige of disease was found within the cranium, but the heart was the seat of the most intense inflammation pervading both the pericardium and the muscular substance. Four or five ounces of turbid serum with flakes of coagulable lymph floating in it were found in the cavity of the pericardium, which had its internal surface covered in various situations with a thin layer of reticulated lymph. Thus far there were the evidences of acute inflammation of the pericardium at an early stage. There was no adhesion of the opposite surfaces: the lymph and the serum had been effused together, and the serum had partially washed away the lymph as it was deposited. Further, when the heart itself was divided, the muscular fibres were dark-coloured almost to blackness, loaded with blood, soft and loose of texture, easily separated and easily torn by the fingers; and at the cut edges of both ventricles small quantities of dark-coloured pus were seen among the muscular fibres. The internal lining was of a deep red colour without any effusion of lymph."

There is good reason to believe that the Cerebral attacks, to which we have been alluding, are often associated with a vitiated state of the blood, in connection with granular disease of the Kidney. Hence the importance of watching the state of the urine.

Supposing, however, that the patient has escaped, or that he has entirely recovered from, all the casualties which we have been mentioning, and that nothing but a very inconsiderable cardiac lesion,—the slightest narrowing, for example, of one of the arterial orifices, or the smallest deposit on one or more of the valves—is left behind, it must be still borne in mind that he will remain extremely liable to a relapse of Carditis from apparently trifling causes. An accidental exposure to cold, a single act of intemperate indulgence, or the putting forth of some unusual bodily effort may soon rekindle the mischief, and thus most seriously aggravate the amount of lesion that already exists. It is, indeed, this very liability to the recrudescence of the primary disease that constitutes one of the most serious features in all cases, where there is reason to believe that the heart has once suffered from rheumatic inflammation.

"Remember," says Dr. Latham, "acute rheumatism is (if we may so speak pathologically) the great parent root of inflammations of the heart. It is also undoubtedly, one of those diseases for which men are found to have a constitutional proneness. When it has been once suffered early in life, there is a fearful likelihood that it will be oftentimes suffered again. Moreover, the first attack is generally the type of every attack which is to follow. They may not all be equally severe, but they will all take the same course, and involve the same structures. If the first involve the heart, so, probably, will they all. Thus, the thought of a healthy child first seized with acute rheumatism is full of sorrowful forebodings. Its heart is very likely to be inflamed, and it may die: but, whether it die or not, its heart is very likely to be damaged for life. Having had acute rheumatism once, though it may perfectly recover, it is very likely to have it again; and, whenever it again has acute rheumatism, it is very likely again to have inflammation of the heart as its accompaniment." P. 29.

Now, in reference to these second and third attacks of Carditis, it is of especial importance to bear in mind that the diagnostic symptoms can never be so satisfactory and conclusive as in the first attack of the disease; nay, that they may be even so obscure as to be extremely liable to be entirely overlooked.* Nor are we surprized at this circumstance in reference

---

* This remark holds true of other organs besides the Heart:—

"It is a general truth, never formally declared perhaps, but well worth our notice, and of great practical importance, that organs must be previously sound to show clearly the nature of the injury or malady which they suffer, and that, in to the heart, when we consider that the organ was not completely sound, and consequently that the auscultatory characters of its sounds were not normal, when the new inflammatory invasion supervened. If there was adhesion of the Pericardial surfaces left by the preceding attack, there may be no exo-cardial or attrition sound heard at all; and, with respect to any fresh deposit on the Endocardial surface, the only effect of this on the auscultatory phenomena will be merely to aggravate the previously-existing murmurs, but not to induce new ones. "There is the permanent murmur of the old unsoundness, and the recent murmur of the new disease; but how much is due to the old, and how much more to the new, is too delicate an affair for the nicest ear to discriminate." How pregnant with good sense, aye too, and with the soundest medical logic, are these reflections of our author!

"After all, then, you will observe, that, for the actual presence of this secondary inflammation in any case, and for our guidance in treating it, we have only the warrant of conjecture. It is most true.

"But there is such a thing as sober conjecture, as well as sober certainty. And diseases are treated, and cures are achieved, and lives are saved, as often under the guidance of one as the other. Such conjecture, however, is altogether different from the arrogant guess-work, which has no basis of action, and which succeeds once and fails twenty times, and knows as little why it succeeds as why it fails.

"The conjecture which should guide the physician, is rigorous, and calculating, and honest. It acts strictly by rule, and leaves nothing to chance. It does not absolutely see the thing it is in quest of, for then it would no longer be conjecture. But, because it does not see it, it ponders all its accidents and appurtenances, and, noting well whither they point, it takes aim in the same direction, and so oftener hits the mark than misses it. And succeeding thus, it knows why it succeeds, and it can succeed again and again upon the same terms.

"Next to knowing the truth itself, is to know the direction in which it lies. And this is the peculiar praise of a sound conjecture. P. 54.

Inconclusive, however, as the diagnostic symptoms of second or third attacks of Carditis must be acknowledged to be, it will be sufficient for the wise physician to know that, with every fresh accession of Rheumatism in a person who has once suffered, there is almost invariably such an increase of uneasiness and palpitation of the heart, of dyspnœa, and præcordial anxiety, as to make him keep a strict watch upon the seat of the chief mischief.

Dr. Latham has very convincingly shewn, in his former volume, that Pneumonia and Pleurisy are often associated with the first attack of Carditis in rheumatic cases. We need scarcely say that the same complications are common attendants upon all relapses of the heart-disease.

---

proportion as they are unsound, they are spoiled for giving true expression to the ills which afterwards befall them. The brain, the lungs, the kidneys, the abdominal viscera, being previously sound and healthy, proclaim themselves inflamed at once. But the brain, with a clot of blood lodged within it, tuberculated lungs, granulated kidneys, a scirrhous stomach, an ulcerated bowel, have their functions and sensibilities in utter disorder and confusion, and are not in a condition to give requisite notice of a new inflammation. A broken instrument is ever out of tune: whatever key you touch, you can never bring out the right note corresponding with it." P. 33.

His remarks on the *treatment* of these secondary attacks of Cardiac Inflammation have chiefly in view to guard the physician against pushing general blood-letting too far. Although the action of the heart and arteries be tumultuous and violent, and the thoracic anxiety and oppression be great, large depletions of blood are seldom, if ever, required. Even mercury is not *commonly* necessary, in his opinion, in the majority of those cases of secondary carditis. The rule, which he gives for its exhibition, is this :—

"Leeches applied to the region of the heart will, by the immediate effect which they produce, test the sort of inflammation you have to deal with, and show whether any and what other remedy will be needed in counteraction of it. If they at once afford marked relief, they thus denote both that the inflammation is easily controllable, and that they, without the aid of any other remedy properly antiphlogistic, will be able to control it. And so it will tnrn out in the majority of cases. But if they afford no marked relief at once, or, still more, after their repeated application, then they plainly proclaim the inflammation beyond their power to cope with, and they call for the help of mercury (as at first) to withhold it from a fatal issue: but this does not often happen." P. 59.

We think that Dr. Latham is rather chary in his use of Mercury in the cases alluded to. His very admission, that fresh and progressive deposits of coagulable lymph on or within the heart may take place without the presence of any very well-marked symptoms at the time, might rationally suggest a somewhat more energetic practice. We are by no means friendly to the active administration of this potent medicine—more especially in the commonly-adopted form of calomel—whenever there is reason to suspect recrudescence of Rheumatic Carditis; but the moderate use of some of the milder preparations, as of the *hydrarg. c̄ creta*, in combination with the *soda carb.*, or the inunction of the mercurial ointment, only very gently to touch the gums, should, in our opinion, be seldom omitted. If we employ the mercurial inunction, the Hydriodate of Potash may be advantageously exhibited internally, at the same time. The application, moreover, of one or two blisters on the cardiac region is always advisable. We have again to express our surprise that so experienced a practitioner as our author should never make any reference to the state of the urine, as affording a very useful guide for determining the proper *duration* of the antiphlogistic treatment to be adopted. As long as this excretion is high-coloured, or exhibits a tendency to lateritious deposit, we may feel assured that the patient should be treated by a cooling antiphlogistic—mild though this may be—regimen. The use, too, of alkaline diuretics, to which Colchicum may be generally added with much advantage, should seldom be omitted.

The organic lesion induced by Endocardial Inflammation may vary so much in point of seriousness as well as extent, that we cannot be surprised at the different effects produced upon the general health of different patients. In some, the only evidence of their having had an attack of endocarditis is the persistence of an endocardial murmur, notwithstanding the perfect integrity of their health, and their capability of active and even of laborious exertion. In others, although their general health is perfectly good, there is, in addition, a tendency to palpitation of the heart, and to a certain degree of dyspnœa, whenever they exert themselves much, as in running, mounting up stairs quickly, or lifting any heavy weight ; mental excitement, too, will have the same effect. Now, we may reasonably infer that the only injury, left behind in these two sets of cases, is a slight lesion—varying in point of degree—in some part of the valvular apparatus of the heart. In a third set of patients, besides the admonitory symptoms just alluded to, the heart is found *always*—whether the individual be at rest or not—to beat with greater force than it was wont to do before the attack. When this is the case, there will be reason to suspect that hypertrophy—with or without dilatation—of the muscular substance of the heart, may have commenced. Still, life may be prolonged for many years without much increase of the mischief, provided the person be not exposed to the operation of those exciting causes which aggravate all cardiac diseases, acute and chronic.

" Taking the three descriptions of cases in their order, I believe," says Dr. Latham, "it to be the tendency of each to pass progressively onward into the others. The endocardial murmur left by acute endocarditis may be simple and alone, and so it may remain for years, but it is ever apt to have a palpitation added to it. The palpitation accompanying the murmur may be occasional only, and so it may continue for years; but, in the mean time, it is ever ready to become permanent. The permanent palpitation may remain for a while moderate in degree, but it is always tending to become greater and greater. Of these three conditions, then, the best that experience allows us to hope is, that each may remain stationary: for their changes are never retrograde, but always progressive and always for the worse. Each condition becomes worse as it is converted into the other, and the condition of permanent palpitation passes on to new results, and to the final and fatal event." P. 97.

It is the consideration of this tendency, however slow and gradual, to a progressive aggravation of the cardiac lesion, which will make the wise physician never fail to instruct and earnestly admonish every patient in whom a persistent endocardial murmur, after a rheumatic attack, is discoverable, to guard himself as much as possible against those influences which are well known to inevitably accelerate the progress of all structural changes of the heart.

There is no sure and constant auscultatory sign or indication of byegone Pericarditis, even when the adhesion between the pericardial surfaces is loose and extensive, as there unquestionably is in a great majority of cases of Endocarditis. It appears to be highly probable that, whenever the pericardium has once been the seat of decided inflammation, it seldom or never resumes its complete or original integrity. The amount of change may, indeed, be inconsiderable, if the attack has been single and not very severe; but nevertheless *there* it is. That a great tendency often exists to the exudation of lymph on the pericardial surface is proved by the frequent occurrence of loose slender adhesions between its opposite faces, or between the pulmonary artery and the aorta, even when there is no record of the person having ever been suspected of having had any cardiac disorder. " Some of these (loose slender adhesions) Mr. Paget never fails to discover, wherever there are white spots upon the heart, and from the coincidence of the two, he has drawn the sound conclusion that both are the effect of inflammation; inflammation, however, of which there are commonly no traces in the history of men's lives, to match these sure and authentic ones met with after their deaths."

But a much more serious organic change is sometimes found to have taken place, without its having been evidenced by any symptoms during life ; viz, the Adhesion of the opposite Pericardial surfaces over the entire extent of the heart. True ; the previous history of the person may not be accurately known ; but of this we may be so far certain, that there was nothing in his case to make even the most enquiring physician suspect the existence of any cardiac lesion, while treating him perhaps for disease of another organ.

"It is a question with me then after all, what are the consequences which naturally result to the functions and structure of the heart from simple adhesion of the pericardium. For I have not facts enough to appeal to of the sort which are required to settle it. Pericarditis indeed is common enough; but not simple pericarditis. The original disease is oftener a complex of pericarditis and endocarditis than pericarditis alone; and the original unsoundness a complex of the partially repaired effects of both. Hence whatever detriment the heart is afterwards found to suffer in its functions and organisation, it is difficult to make sure either how much is due to each, or whether the whole may not be imputed to one; how much the thickened valve produced, and how much the adhesion of the pericardium ; or whether the thickened valve may not have been exclusively the source of all the mischief, and the adhesion of the pericardium altogether blameless from first to last." P. 112.

When the adhesion of the pericardial surfaces exists not universally, but only at different points, leaving intervening spaces of unattachment, these spaces (or *loculaments* as a Botanist might call them) may become, from a subsequent attack of pericarditis, the seat of a purulent effusion ; and thus the heart may be found, upon dissection, to be apparently surrounded with a number of separate little abscesses.

The adventitious tissue, that unites the opposite pericardial surfaces, is found to vary exceedingly in point of density and thickness. Sometimes it is so attenuated that the two laminæ seem to be merely incorporated with each other, without any intermediate substance ; while, in other cases, the uniting medium has been found to be half an inch, nay more than an inch, in thickness. The nature and appearance too of this substance may vary a great deal.

"Its texture sometimes laminated like the coagulum of an aneurismal sac, red or tawny near the heart, and pale or white more remote from it, sometimes of a mixed consistence, in part almost liquid and purulent and in part solid or tuberculous. Or the adventitious substance has been of one uniform texture, either so like muscle as to be at first mistaken for the fleshy substance of the heart itself, or so far firmer than muscle as to resemble flesh hardened in brine, either much paler than the heart, or much redder from being deeply injected with blood. This tough flesh-like substance may occupy a portion only of the surface of the heart or the whole of it. I have seen it opposite the right auricle, while every where else the pericardium has closely adhered with little intervening medium, and I have seen it enveloping the entire organ and forming round it (as it were) another case of muscle. And then, if (what often happens) the muscular substance of the heart itself be augmented, a strange spectacle is disclosed on dissection. There is an enormous mass displacing the lungs and leaving nothing visible in the entire front of the chest but itself." P. 116.

Now all this amount of most disorganizing change may unquestionably be traced back to an attack of simple Pericarditis, which may have occurred some years before. Successive accessions of inflammatory disease (masked and unrecognized although these have been) have only served to cause successive depositions of fresh layers of lymph ; these layers indeed varying in point of colour and consistence, from causes which we do not understand. When the connecting substance exhibits a uniform colour and consistence throughout, it seems not improbable that a process of progressive interstitial deposition of new matter had been going on for a length of time, without the supervention of any fresh or distinct attacks of pericardial inflammation. But, in whatever way we choose to explain the formation of the morbid change, this one thing is certain, that a most serious amount of mischief may take place on the pericardial surface of the heart without producing any marked aggravation of the cardiac distress, which was unquestionably attributable to the first attack of pericarditis.

From the consideration of the diseases of the lining membranes, the Endocardium and the Pericardium, of the heart, we now pass on to that of the chief morbid changes to which the Muscular Substance of this vital organ is liable ; as Suppurative Inflammation,—of which two cases are recorded ; one of these we have related in a preceding page, the other is that detailed by Mr. Salter in the 22nd Vol. of the Medico-Chirurgical Transactions—Attenuation and Softening with or without aneurismatic Dilatation of one or more of the cardiac cavities, Hypertrophe, Fatty Degeneration, &c.

Whether it is correct to speak of the *flabby, attenuated, and lacerable state of the heart* as a result of chronic inflammation may be fairly questioned. Certainly, in the cases related by Dr. Latham, there is not a shadow of evidence to make one believe so. In both instances, the fatal attack began with vomiting and diarrhœa ; the patients recovered for the time, and there was nothing to excite the suspicion of approaching death. But the weakness produced by the intestinal disorder seems to have so aggravated the long existing, although not suspected, disease of the heart, as very seriously to have interfered with the due performance of its functions. Perhaps it will be interesting to give the description of the condition of the left Ventricle, the chief seat of the lesion, in both cases.

In the first, " that portion of the left ventricle already mentioned, which in its external aspect gave suspicion of an abscess, presented the following conditions of disease. There the heart was so attenuated as not to exceed the breadth of a half-crown piece, and rupture or ulceration preparatory to rupture was in progress. The internal lining was destroyed, and to the rough surface that it left a large irregular-shaped clot of blood was adherent. What remained exterior to the clot had lost all cognizable organisation ; it hardly cohered together and was torn like wet paper.

The aorta throughout its course within the chest (for so far only it was examined) was dotted with little earthy and atheromatous deposits."

In the second, " the left ventricle was very capacious and its walls thicker than natural, except at one circumscribed space. This was between the two large carneæ columnæ. Here, at the expense of the muscular substance which had entirely disappeared, a cavity was formed large enough to contain half a walnut. The thickened lining membrane was here united by lymph to the serous covering of the heart, and both together formed its external boundary. It was diaphanous, and served for the only barrier which prevented the blood flowing from the ventricle into the cavity of the pericardium. There was no laminated coagulum in the aneurismal pouch."

In both instances, rupture was, as our author remarks, only just anticipated by death ; the immediate cause of which was probably a paralytic incompetency of the heart to continue its contractions. In some cases, as is well known, actual laceration of the organ takes place ; and then, we need scarcely add, the dissolution is almost immediate.

And here we may allude to a very important fact in the history of very serious organic lesions of the muscular substance of the heart; we mean, the circumstance of the arterial pulse being often but little affected. Cases of very great dilatation, accompanied with softening and attenuation of the ventricular parietes, not unfrequently occur, in which the pulse retains a regularity in point of frequency and force perfectly remarkable. We need not say how apt this circumstance must be to mislead the unsuspecting physician, more especially if he is not in the habit of exploring the state of the chest. In Mr. Salter's very remarkable case, already alluded to, one of its most striking features was the regularity of the arterial pulse and of the heart's action, until within the last 48 hours of life ; and yet there cannot be a reasonable doubt but that the substance of the left ventricle had been the seat of Suppurative Inflammation for two or three weeks at least before death ! Equally striking was this circumstance in the very interesting case of Rupture of the *septum cordis* narrated by Dr. Latham. Within 17 hours of death, at the time, too, when the patient was deadly pale and every part of the surface was as cold as marble, it is expressly stated that " the pulse was of a good strength, and the heart was contracting regularly and forcibly." Ten hours subsequently, no pulsation could be felt in the arteries, while the heart was perceived by the ear to move, but not by the hand : in this state the patient survived for seven hours.

The prolongation of life for several days, and even for a week or two, when dissolution has seemed to be almost impending, is another circumstance in the history of cardiac disease, that cannot have failed to have struck the attention of the practical physician. We have, more than once, left a patient over night in such an alarming condition of imminent asphyxia that we did not expect to find him alive next morning ; yet he has lived for a good many days afterwards.

We have now to direct our reader's attention to a chapter in the history of cardiac diseases, which has hitherto been scarcely so much as mentioned, and therefore calls for a somewhat lengthened notice ; we allude to what our author terms " Shocks of the Heart," in other words, traumatic injuries of one or more of the valves of the heart, induced by any violent bodily exertion, and laying the foundation for future hypertrophy, or dilatation, or both these morbid states of its muscular substance. Dr. Latham quotes the following case, communicated by Dr. Bence Jones, as a type or exemplar of such cases.

" A stableman, twenty-eight years of age, was admitted into St. George's Hospital. He was suffering, and had suffered for twelve months, severe palpitation of the heart, and was able to mark distinctly the moment of its commencement. It was one day just after running a horse down the yard to show off his paces to a purchaser. He had never had acute rheumatism. His lips were blue, his breath short, and his left side painful. He had a dry cough. His bowels were confined and his urine free. It was ten weeks before his admission that his cough and dypsnœa had begun to be particularly distressing. Auscultation found dulness in the præcordial region over an extent of four inches square, the heart's impulse increased and its first sound prolonged with a low blowing (endocardial) murmur over the aortic valves, and its second sound indistinct. He was bled three times under the urgency of his cough and dyspnœa. These however continued to increase. Five weeks after his admission his legs became œdematous, and in two weeks more he died.

"On examination after death, three pints of fluid were found in the right pleura, and the heart enormously large. In length it reached from the second to the eighth rib, and across the base of the ventricles it measured six inches. The left ventricle was moderately hypertrophied and very largely dilated. The mitral valve was healthy, and the aortic was slightly thickened, and moreover had suffered rupture of a peculiar kind. One of its septa was torn away from its attachments, and thus two of its pouches were reduced to a single irregular one. The right ventricle was dilated, but both the auricles preserved their natural state. In the ascending aorta and in its arch there were atheromatous deposits. The liver was very large, and the spleen and the kidneys were healthy." P. 194.

Now any violent effort or fatiguing exercise of the body may give rise to the mischief that was found in the preceding case. Many a youth lays the foundation of cardiac disease in feats of rowing, leaping, boxing and such like sports. The same thing is not unfrequently the case with horses that have been galloped severely : indeed, immediate rupture of the heart, in a horse previously quite sound and healthy, has been known to be caused in this way.

Whether in "shocks of the heart," followed by tendency to palpitations and other symptoms of disordered heart, there is always a rupture or other lesion of one of the valves, it is not possible to say. It is sufficient to know that, unless great precautions—in the way of quietude, low diet, and (it may be necessary) sanguineous depletion—be used for a very considerable time after the injury, there is good reason to apprehend the supervention of hypertrophic enlargement or of dilatation of the heart. Dr. Latham narrates at length the case of one of his friends, in whom the symptoms of a "shock of the heart"—viz: excessive impulse, and pain in the cardiac region—followed upon a violent collision against another person in the street, while running at full speed. He was treated by the late Dr. Baillie, who bled him largely. It was only after the lapse of some months that he was allowed to return to his avocations. By this time, he had lost his *constant* palpitation. But for a few years, it was wont to return painfully upon occasions of excitement. At length he lost it altogether; and lived 25 years after the shock and perilous illness that was the consequence, actively engaged in a laborious profession. Now, is it at all improbable that, had not the judicious and long-continued precautionary treatment been followed in this case, hypertrophy and dilatation would have ensued? At all events, there is surely sufficient reason to believe that such consequences have followed upon severe bodily injuries, in persons who had never been suspected of having any tendency to, or degree of, cardiac enlargement.

In the last number of the Edinburgh Monthly Journal of Medical Science, Dr. Quain has published a very interesting paper, entitled "Cases illustrating the injuries to which the Aortic Valves are liable during muscular efforts." The first case recorded is the following :

A man, 26 years of age, who was in good health at the time, and had never suffered from rheumatism, palpitation of the heart, or shortness of breath, was suddenly seized (August 1843), while using a sledge-hammer, with a most distressing sensation in the region of the heart, which compelled him to give over. He complained of "an uneasy shaking of the heart," dyspnœa, and what he called "a noise up his ehest and neck, and in his ears," which prevented him from sleeping. For a week subsequently, he continued at light work; but, becoming worse, he came under Dr. Quain's care at the University College Hospital. "There were then very distinct evidences of imperfection of the aortic valves; in the situation of these valves, and replacing their sound, was heard a loud ringing musical murmur—the first sound was also accompanied by a murmur in the same situation, but much less loud: there was present the peculiar diastolic or regurgitant pulse."

Five weeks from the date of the accident, it is recorded that "there are now very evident signs of Enlargement of the heart. The dulness on percussion over its site and its impulse are extended, and the force of the latter is increased. The murmur is so loud that it can be heard at several inches distance from the aural end of the stethoscope, and the diastolic pulse is so marked as to give a very peculiar appearance to the course of the superficial arteries."

Three months later, when trying to do some heavy work, the patient again became conscious of a change in the heart's action; and it was now discovered that "the loud ringing murmur with the second sound, and the slight murmur with the first, were both replaced by the ordinary double bellows-murmur."

The poor fellow lived for two years after the accident, occasionally able to do some light work, but every now and then having attacks of bronchitis, dyspnœa, palpitation of the heart, and *angina pectoris*. The signs of Hypertrophy of the Heart increased; the loudness of the murmur diminished, but its character remained the same. In July 1845, he became anasarcous; the physical symptoms were nearly as before, regurgitation through the jugular veins being now very evident. On the 10th of August he died suddenly, in one of his attacks of dyspnœa.

*Dissection.*—"On the chest being opened, the heart, enveloped in the pericardium, was found to have encroached much on the situation of the inferior lobe of the left lung. There were traces of old disease of the apices of the lungs; the bronchi were thickened, and the mucous membrane congested. All the cavities of the heart were enlarged and filled with blood. The arch of the aorta was somewhat dilated. The heart weighed 22½ oz. The chief disease was found at the entrance of the aorta; here it was noticed that the conjoined attachments of two of the valves to the aorta had been separated from the wall of that vessel, and thus those valves were allowed to drop below the level of the third, which retained its connexions. In the drawing, *a* indicates the junction between the valves, *b* is the point at which the separation has taken place; here the wall of the vessel was raised into a superficial elevation about one-third of an inch in length and one-fourth of an inch across. The margin of one of the valves was everted slightly, and studded with small granulations, represented at *c*. It seemed as if a small strip of the living membrane had been torn off at this point. On trying the valves with water before the vessel was cut open, they were found to be quite inefficient, not so the pulmonary."

The most remarkable feature in the preceding case is the rapidity with which the very great hypertrophic enlargement of the heart (it was nearly three times its ordinary size) took place; an alteration that serves to explain the improvement which occurred in the patient's symptoms: the increased muscular power of the heart overcoming in some degree the insufficiency of its valves. Another peculiarity, that is worthy of notice, was the proneness to Bronchitic attacks—a result, no doubt, of the irregularity in the circulation of the blood.

Besides the foregoing case, Dr. Quain mentions two others, in which the primary cardiac lesion—a rupture of the valves—seemed to have been originated in a somewhat similar way.

A porter, in good health, endeavoured, while highly excited, to force open a door with his shoulder. "He was seized at the moment with an oppressive sensation in his chest, and, when examined with the stethoscope, the aortic valves were found to be imperfect. His breathing became embarrassed, his heart hypertrophied, and his body anasarcous. He died in about eighteen months from the date of the accident. The imperfection of the aortic valves was found to depend on the convex (inferior) margin of one of them being torn from its attachments, resembling thus a pocket which had been ripped or torn at the end. The heart was much hypertrophied."

The last case occurred in a man, 54 years of age, who has never had rheumatism nor any disease of the heart, of which he was aware. While carrying a very heavy load of timber on his back, and in the act of stooping to admit of its removal, he was suddenly seized with so severe a pain in the region of the heart, that he was obliged to let fall his load. Palpitation and dyspnœa commenced at the same time; and at night, on lying down, he heard a noise which has continued to distress him ever since. When he came under Dr. Quain's observation, five months after the accident, he presented strongly-marked signs of imperfection of the aortic valves and of hypertrophy of the heart. " 'Both sides of the chest are equal in circumference, showing therefore an increase of the left. The apex of the heart is *seen* and *felt* to beat below the seventh rib. The motion has an undulatory appearance. The strength of the impulse is not proportioned to its extent; a distinct vibratile thrill is *felt* over the entire region of the heart, also over the right carotid and subclavian, but to a much less degree in the left. The diastolic pulse is seen and felt. On *percussion*, dulness in the region of the heart is found to extend upwards from the seventh rib, in a line with the right shoulder, 5 inches; vertically from the third costal cartilage, $3\frac{3}{4}$ inches; and directly across the centre of the heart, $3\frac{1}{4}$ inches. By *auscultation*, the second sound is not heard, but its place is taken by a loud musical murmur, which is heard all over the chest, but most distinctly at its upper part, and over the base of the heart. There is also a murmur, but much less loud, with the first sound; it is heard in the same situation, but more distinctly in the carotids than that with the second.' His urine is slightly albuminous." He has derived relief from the use of cupping over the region of the heart, and of sedatives, &c. He still survives.

These cases of Dr. Quain are very instructive. Dr. Latham mentions an interesting one, to shew that a violent fit of Rage may (very probably)

be the cause of the same valvular lesion which, we have seen, may be produced by a Physical Shock.

A man, in a state of the greatest excitement, seized a knife, and was just plunging it into his own throat, when his wife, with whom he had been quarrelling, rushed upon him, disarmed him, and disappointed his purpose. Some neighbours came in and secured him until his rage had burnt itself out. But from that day he had always been sensible of a palpitation of the heart, which had gradually increased until it incapacitated him for work. Then he became dropsical, was admitted into the hospital, and soon died. All was the work of not many months.

We have now seen that whatever serves to interfere with the smooth and easy play of the heart's action, has a tendency to induce Hypertrophic Enlargement of this vital organ. Such being the case, it is not wonderful that the lesion in question is a common consequence of the existence of any obstruction to the free passing of the blood through the great arterial vessels, and consequently to its easy exit from the ventricular cavities. Hence narrowing of the Aorta, in any part of its course, almost inevitably induces hypertrophy and dilatation of the heart; except, indeed, the mass of blood becomes so much reduced as that the deficient quantity of the permeating fluid makes up and compensates for the abridged calibre of the vessel through which it has to pass. It is, doubtless, in this way alone that we can account for the fact, that the right cavities of the heart are very rarely indeed found to be enlarged in cases of Phthisis, (in which disease there must be most serious impediment to the circulation through the pulmonary arteries) although such enlargement is of frequent occurrence in all the forms of Asthma—in other words, in all diseases in which embarrassment of the breathing, whether occasional or more permanent, is not accompanied with injury of the nutritive functions, and therefore with wasting of the body.

Deformity of the Chest, resulting from curvature of the spine, may become, in point of its effects upon the heart, equivalent to constriction of the aorta, to solidification of the lungs, or, in short, to any other morbid state that occasions a mechanical impediment to the free circulation of the blood from the centre to the extremities ; and thus be the producing cause of hypertrophic enlargement. But, besides the causes already mentioned of this frequent morbid condition, there are others of a less obvious character, and therefore more likely to be overlooked. The following passage points to one of these.

"The coincidence of disorganization of the heart, especially of its hypertrophy and dilatation, with the marks of chronic disease extensively diffused throughout the arterial system, is very common. The internal lining of the arteries, here and there, in various situations, and upon the whole to a great extent, has lost its transparency, and become a little thickened, and dotted with cartilaginous and atheromatous and bony deposits; but nowhere has its change of structure been such as could be thought capable of producing injury simply by mechanical impediment. And this may be all that is found in the body to account for the heart's unsoundness. But this mere beginning of disease in the arteries, which is indeed a small matter when we see it in single blood-vessels, becomes a great matter, and capable of great effects when it spreads itself throughout the body. It may well be conceived enough to make itself felt by the heart.

"In looking over such records of cases as I possess, it is remarkable in how large a proportion of them I find this condition of the arteries coincident with hypertrophy and dilatation of the left ventricle. And this, I have said, may be all that is found in the body to account for the heart's unsoundness. But oftentimes there is this and much more than this. We see that the disease of the arteries has reached a more onward stage, and made larger and more extensive deposits of cartilage and atheroma and bone, while the liver and the spleen and the kidneys are found enlarged and granulated; and the transparent membranes, as the pleura and peritoneum, are thickened and opaque. These are evidences and effects of chronic inflammation, and have a pathological connection one with another. And it is strange, if they have not also a connection with the disease diffused throughout the arteries; and it is strange, moreover, if they have not all a connection with the hypertrophy and dilatation of the heart." P. 226.

Still we must remember that there is no direct evidence to shew that the affection of the Heart has been the result or *effect* of disease of the Arteries; nay, the very converse may be nearer the truth. As yet, therefore, we must be content to know that the two morbid states are not unfrequently coincident; and that the existence of such a complication may be the cause of heart-disease being very much more intractable than a more aggravated form of the organic lesion when the arterial system remains unaffected. Another occasional coincidence, if not a cause, of certain affections of the heart, that is enumerated by Dr. Latham, is granular disease of the Kidney, indicated by the albuminous condition of the urine. As with the diseases of other viscera in this cachectic state of the system, so with the heart, it is more than probable that the depravation of the circulating fluid is the agency or medium by which Bright's disease operates upon the functions—and it may be on the structure too—of this vital organ. It seems to us very doubtful whether organic disease of the heart can ever be fairly attributed to granular degeneration of the kidneys as a cause. It is more probable that the latter may be gradually induced by the former, in consequence of the stagnation and congestion of blood in the renal structure, arising from an obstruction to the free return of the venous blood into the right ventricular cavities. However this may be, there is no doubt that it is of great importance, more especially in a prognostic point of view, to watch the state of the kidneys, as indicated by the urine, in all diseases of the heart. The same thing may be said of the Liver. The increase of distress, produced by congestion of this very sanguineous viscus, cannot fail to strike the attention of every practitioner.

In forming our *Prognosis* in a case of Valvular Disease or other organic affection of the heart, we must take into account not only the suspected amount of the lesion of the valve or valves, but also the probable cause of the disease, as well as the character of the patient's constitution and his general habits and mode of living. When we have reason to believe that the malady has been the result of rheumatic endocarditis, the prognosis as to its duration will, *cæteris paribus*, be more favourable than when it has proceeded from other causes. The reason of this is obvious. By appropriate medical and dietetic treatment, the tendency to rheumatic disorders may unquestionably be very much subdued; and, as the general constitutional health may be sound, the great object of the physician is merely to prevent the recurrence of a rheumatic attack, and to guard his patient against those errors of exercise and so-forth, which are so hurtful in all cardiac maladies without exception.

"On the other hand, advanced life and a cachectic aspect, and the known habits and ailments of intemperance, or some bad hereditary disposition strongly marked, or frequent attacks of some constitutional disorder, such as gout, or rheumatism, or gravel, would hardly suffer us to hope that the disease was single and solitary in the mere valve of the heart which it had injured, (though auscultation did not testify to more,) but would rather lead us to fear its universality in the whole arterial system. Here indeed we fear more than we know. But this is a rational fear." P. 241.

With respect to *Treatment*, it can scarcely require to be stated that our hopes of relieving our patient are to rest almost entirely upon the steady observance of a judicious hygienic regimen, and the regulation of the general health. The management of a case of organic disease of the heart can only be safely conducted by the scientific physician; the mere prescriber is more likely to do mischief than good. No two cases can be treated in exactly the same manner. We perfectly agree with our author that the true nature of his disease should never be concealed from the patient. On the whole, it is a great mistake—and one too that may be a cause of regret ever afterwards—to suppose that the alarm, upon being made acquainted with the truth, may produce serious aggravation of the existing malady. All depends upon the manner in which the communication is made, and upon the remarks which accompany it. Woe betide that man who, whether from indifference to the feelings of others, or from a mere insolent parade of technical knowledge, cruelly proclaims the hopelessness of a malady, without one expression of sympathy or guarded encouragement to the poor sufferer!

On the subject of the treatment of Hypertrophy, there are some judicious and useful observations in the 31st Lecture. That many cases are erroneously set down in practice as examples of this lesion, merely because there is an abiding increased force in the impulse of the heart, is but too true; and the consequences of the mistake are often much to be deplored, as leading to groundless alarm as well as a most unnecessary activity in the measures adopted. The following extract contains the most valuable portion of Dr. Latham's remarks on the subject:—

"Impulse of the heart, taken alone, however great and however extensive it may be, is not a sure physical sign of hypertrophy. Hypertrophy indeed cannot exist without excess of impulse, but excess of impulse can exist without hypertrophy. When the impulse of the heart is excessive, and at the same time its sounds are obtuse, muffled and indistinct, and the præcordial region presents a larger space than natural which is dull to percussion, then the signs of hypertrophy are complete. And hypertrophy so sure and unquestionable was never cured within my experience. But when the impulse of the heart is in excess, and at the same time its sounds are as loud and clear as ever, or louder and clearer still, and the whole præcordial region is quite resonant to percussion save the small space which is naturally dull, then the signs of hypertrophy are incomplete. Yet if this be enough to constitute hypertrophy, I have seen and treated it successfully in a hundred instances. But in the mean time I have not thought that I had to do with any such affection or ever claimed the least credit for curing it.

"Cases of mock hypertrophy of the heart are indeed very numerous. Young persons at the prime of life are especially the subjects of it. They are often plethoric and often sedentary, and can assign the origin of their complaint to no particular time and to no particular exciting cause. In them, the excessive action of the heart is doubtless owing to a rich and redundant blood; and the cure of their simulated hypertrophy is effected by depletion and abstinence, and the gradual exchange of indolent for active habits. These are easy cases to deal with.

"Again, young persons are the subjects of it, but they are often pale and thin and dyspeptic and very sensitive, and inactive from mere debility and nervousness. In them the excessive action of the heart cannot be ascribed precisely to any one thing. The stomach and the nerves and the blood itself are all disordered, and they all are sources from which injurious influences may spring up and travel to the heart; and they all have probably their share in producing the simulated hypertrophy. Being so produced, its cure can only be effected by varied methods of treatment and after a long time, and often not until the constitution has undergone some of those changes which belong to stated periods of life. These are by no means easy cases to deal with.

"Again, young persons are the subjects of it, but they are often neither florid nor pale, neither too full nor too empty of blood. They have no complaint that they can tell you of, and none that you can make out, except an inordinate impulse of the heart; an impulse great enough for any amount of hypertrophy, and constantly present, and admitting of severe aggravation, and ever attended with pain, while the sounds of the heart are still loud and clear, and the præcordial region is still duly resonant.

"These cases are the most difficult of all to deal with. Yet their treatment seems theoretically to lie within a narrow compass. There are no ailments of other organs to set right with the hope that through them you may reach the ailment of the heart. The heart itself contains within itself the sole indication of its treatment. Abate its violent impulse and all will be well. But bleeding will not abate it. Neither will all the variety of anodynes and antispasmodics. Neither will digitalis. For digitalis cannot be given long enough and largely enough for any fair hope of it as a remedy, without fearful hazard of it as a poison. In truth I know no certain medicine and no certain plan of medical treatment that will abate this impulse. But still I know that the very worst of these cases may get well. I have seen some such and watched them for a time and then have lost sight of them, and cannot tell how they have ended. And some I have seen again after the lapse of years, and found them as bad as ever; and some I have found perfectly well. In these last cases then how has the cure been wrought? Why, it has not been wrought in the way, which would imply a gradual process of bringing down an overgrown structure to its nutural size and dimensions. But it has been sudden and abrupt, without any strict use of appropriate means, and sometimes with an utter neglect of them." P. 252.

One or two of the most useful sedatives of the heart's actions are not so much as even named by the lecturer. Of these, Antimony, in minute doses, is unquestionably one of the most potent. Hydrocyanic Acid is also very useful in some cases. The occasional application of a blister is a good remedy. The same may be said of a belladonna plaster. But, of all external remedies, a seton over the heart is unquestionably the most efficient.

There still remain some important matters to be considered; but the examination of these must be deferred to our next Number, when we shall take occasion to compare the views of the French School on several points with those of our author. Meanwhile, we gladly seize this opportunity of expressing our cordial approbation of these Clinical Lectures. We know of no work where the reader will find so clear and easily-intelligible a view of a difficult practical subject—the Diagnosis of Diseases of the Heart—as in Dr. Latham's two volumes. His interspersed remarks, too, on many questions of general pathology, stamp him as one of the most enlightened writers of the day.

